# Selective IL‐23 Inhibition in Conventional Treatment‐Refractory Pyoderma Gangrenosum: A Multicenter, Retrospective Study

**DOI:** 10.1111/ijd.70430

**Published:** 2026-04-18

**Authors:** Luca Bettolini, Carlo Alberto Maronese, Federica Derlino, Giovanni Genovese, Chiara Moltrasio, Luisa Sarno, Stefano Buffon, Bianca Cei, Giammarco Granieri, Valentina Dini, Marco Romanelli, Vincenzo Maione, Stefano Bighetti, Marina Venturini, Edoardo Cammarata, Paola Savoia, Antonio Di Guardo, Luigi Gargiulo, Ruggero Cascio Ingurgio, Antonio Costanzo, Lorenza Burzi, Claudia Leporati, Nunzia Di Cristo, Nicola Zerbinati, Alex G. Ortega‐Loayza, Angelo Valerio Marzano

**Affiliations:** ^1^ Dermatology Unit Fondazione IRCCS Ca' Granda, Ospedale Maggiore Policlinico Milan Italy; ^2^ Dermatology Department University of Brescia, ASST Spedali Civili di Brescia Brescia Italy; ^3^ Department of Pathophysiology and Transplantation Università degli Studi di Milano Milan Italy; ^4^ Dermatology Unit, Department of Clinical and Experimental Medicine University of Pisa Pisa Italy; ^5^ SCDU Dermatologia, Azienda Ospedaliero‐Universitaria Maggiore Della Carità Novara Italy; ^6^ Department of Health Science University of Eastern Piedmont Novara Italy; ^7^ IRCCS Istituto Dermopatico dell'Immacolata (IDI‐IRCCS), Dermatological Research Hospital Rome Italy; ^8^ Dermatology Unit IRCCS Humanitas Research Hospital Milan Italy; ^9^ Department of Biomedical Sciences Humanitas University, Pieve Emanuele Milan Italy; ^10^ SS Dermatologia, Azienda Ospedaliero—Universitaria SS. Antonio e Biagio e Cesare Arrigo Alessandria Italy; ^11^ Dermatology Unit, Department of Medicine and Innovation Technology (DiMIT) University of Insubria Varese Italy; ^12^ Department of Dermatology Oregon Health & Science University Portland Oregon USA

**Keywords:** guselkumab, IL‐23 inhibitors, neutrophilic dermatoses, pyoderma gangrenosum, risankizumab, tildrakizumab

## Abstract

**Background:**

The therapeutic management of pyoderma gangrenosum (PG) is challenging. Conventional therapies, including systemic corticosteroids and cyclosporine, are limited by incomplete responses, frequent relapses, and cumulative toxicity. Emerging evidence supports a pathogenic role of the interleukin (IL)‐23/IL‐17 axis in PG, suggesting selective IL‐23 inhibition as an appealing therapeutic approach. This study assessed the effectiveness, safety, steroid‐sparing potential, and predictors of response to selective IL‐23 inhibitors in conventional treatment‐refractory PG.

**Methods:**

This multicenter retrospective study included adult patients with refractory PG or intolerant to conventional systemic therapies, treated with selective IL‐23 inhibitors (guselkumab, risankizumab, or tildrakizumab). Clinical assessments were performed at baseline and during follow‐up (1, 3, 6, 12 months, and last observation), evaluating ulcer area, number of lesions, ulcer depth, border wound bed characteristics, Investigator's Global Assessment for PG (IGAPg), and pain intensity assessed by the Numerical Rating Scale (NRS). Longitudinal changes were analyzed using non‐parametric tests for repeated measures.

**Results:**

Selective IL‐23 inhibition in 18 patients was associated with a progressive reduction in total ulcer area, significant from 1 month onward (*p* = 0.0069; all subsequent *p* ≤ 0.0003). The number of active ulcers did not change at 1 month (*p* = 0.41) but significantly decreased from Month 3 onward (*p* = 0.004 to < 0.001). Ulcer depth remained unchanged at 1 month (*p* = 0.41) and significantly improved from Month 3 onward (*p* = 0.01–0.046). Border/perilesional inflammation and wound bed characteristics showed significant improvement at all follow‐up visits (*p* = 0.0008–0.034 and *p* = 0.013–0.046, respectively). IGAPg score showed a significant and progressive improvement over time (all *p* ≤ 0.027). Pain intensity showed a significant reduction during follow‐up (*p* = 0.004 to < 0.001). A significant steroid‐sparing effect was observed, with a progressive reduction in median daily prednisone‐equivalent dose during follow‐up (*p* = 0.002–0.014). Guselkumab and risankizumab showed comparable efficacy (*p* = 0.182). In univariate analysis, baseline ulcer depth was the only predictor of ulcer area reduction (*p* = 0.039). No unexpected safety signals were observed.

**Conclusions:**

Selective IL‐23 inhibition appears to be a well‐tolerated therapeutic option for refractory PG, providing meaningful clinical improvement and a relevant steroid‐sparing effect. These findings support further controlled studies to define the role of IL‐23 inhibitors within PG treatment algorithms.

## Graphical abstract

The clinical effects of selective IL‐23 inhibition in refractory pyoderma gangrenosum, highlighting progressive ulcer healing, reduction in inflammation and pain, and steroid‐sparing effect, supporting the role of the IL‐23/Th17 axis as a therapeutic target.

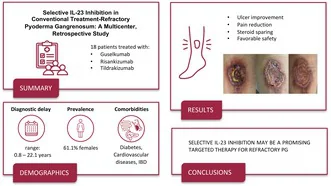

## 1 Introduction

Pyoderma gangrenosum (PG) is a rare neutrophilic dermatosis characterized by painful cutaneous ulcers and frequent systemic comorbidities [1]. Its management remains challenging due to diagnostic complexity, clinical heterogeneity, and the lack of approved targeted therapies. Systemic corticosteroids and cyclosporine are considered first‐line treatments for moderate‐to‐severe disease but are limited by incomplete responses, high relapse rates, and cumulative toxicity, underscoring a substantial unmet therapeutic need [2, 3].

Recent pathogenic insights have placed PG within the spectrum of autoinflammation, driven by a polygenic background and dysregulated innate immunity [4, 5]. Local as well as systemic upregulation of a multitude of proinflammatory cytokines has also been demonstrated, particularly within the IL‐23/IL‐17 axis [6], hinting at a contribution of adaptive immunity to PG pathogenesis and supporting a key role of this pathway in sustaining chronic inflammation and tissue damage. Immunohistochemical analyses have further shown overexpression of IL‐23 and its receptor at the ulcer margins of active disease, accompanied by increased dermal infiltration of IL‐23R^+^ CD4^+^ T cells and elevated expression of neutrophil‐attracting chemokines, including CXCL1, CXCL8 (IL‐8), and TNF‐α. This Th17‐driven neutrophilic feed‐forward loop likely contributes to persistent tissue injury and impaired re‐epithelialization [7].

While anti‐TNF‐α agents and the IL‐12/23 inhibitor ustekinumab have been used off‐label with variable efficacy [8, 9, 10], emerging evidence from case reports and small series suggests that selective IL‐23 inhibition may represent a more targeted and potentially better‐tolerated therapeutic strategy [11, 12, 13, 14, 15].

The present multicenter pilot study evaluated the effectiveness, safety, steroid‐sparing potential, and predictors of response to selective IL‐23 inhibitors in patients with conventional treatment‐refractory PG.

## 2 Methods

This retrospective, multicenter study was conducted across eight tertiary referral dermatology centers in Italy. Data collection covered patients treated between November 2019 and September 2025.

Adult patients (≥ 18 years) with a diagnosis of PG were eligible for inclusion. PG diagnosis was established based on compatible clinical features and histopathological findings, and supported by a PARACELSUS diagnostic score ≥ 10 [16]. Only patients with refractory disease were included, defined as failure, intolerance, or contraindication to conventional systemic therapies, including systemic corticosteroids and/or immunosuppressive agents. All patients were treated with a selective IL‐23 inhibitor (guselkumab, risankizumab, or tildrakizumab) and had a minimum clinical follow‐up of 12 weeks.

Patients were excluded in the presence of alternative causes of cutaneous ulceration, insufficient follow‐up, incomplete relevant clinical data, or concomitant treatment with other biologic therapies during anti‐IL‐23 exposure.

### 2.1 Data Collection

Demographic and clinical data were retrospectively retrieved from medical charts across participating centers. Recorded variables included age, sex, body mass index, smoking status, relevant comorbidities, disease subtype, disease duration, time to diagnosis, anatomical distribution of lesions, and PARACELSUS score. Previous systemic treatments were documented, including corticosteroids, conventional immunosuppressants, biologic therapies, and small molecules, together with treatment duration, reasons for discontinuation, and adverse events.

### 2.2 Treatment Exposure

Selective IL‐23 inhibitors (guselkumab, risankizumab, or tildrakizumab) were prescribed off‐label and administered using standard dosing regimens approved for inflammatory skin diseases or dose/frequency‐intensified regimens. Concomitant systemic corticosteroids were tapered or discontinued whenever clinically feasible.

### 2.3 Clinical Assessment

Clinical evaluations were performed at baseline (treatment initiation) and during follow‐up visits at approximately 1, 3, 6, and 12 months, with an allowed time window of ±15 days, as well as at the last available observation. At each visit, the number of active ulcers, anatomical location, and total ulcer area (cm^2^) were recorded. Overall disease severity was assessed using the Investigator's Global Assessment for PG (IGAPg), which provides a global patient‐level score derived from the integrated evaluation of ulcer depth, border/perilesional inflammation, and wound bed characteristics across all active lesions (Table S1) [17]. Pain intensity was evaluated using the Numerical Rating Scale (NRS; 0–10). Clinical assessments were based on direct examination and standardized clinical photographs acquired according to good clinical practice. Assessments were performed independently by two dermatologists, with adjudication by a third in case of disagreement.

### 2.4 Statistical Analysis

Statistical analyses were performed using IBM SPSS Statistics (version 29.0). Continuous variables were tested for normality using the Kolmogorov–Smirnov test and are presented as means with standard deviations or medians with ranges, as appropriate. Longitudinal changes across multiple time points were analyzed using the Friedman test, while paired comparisons between consecutive visits were conducted using the Wilcoxon signed‐rank test. Comparisons between independent groups were performed using the Mann–Whitney *U* test. Univariate analyses were performed to explore associations between baseline variables and clinical outcomes. A two‐sided *p* < 0.05 was considered statistically significant.

### 2.5 Ethical Considerations

This study was approved by the local ethics committee (approval number: 487_2020). The approval specifically covered the retrospective collection and analysis of clinical data, conducted without deviation from routine clinical care. All patients had previously provided written informed consent for off‐label treatment and for the use of their anonymized clinical data during standard clinical visits. All requests for off‐label prescription of selective IL‐23 inhibitors were submitted to the National Center for Rare Diseases Networks and approved by the competent local ethics committee. The study was conducted in accordance with the principles of the Declaration of Helsinki, Good Clinical Practice guidelines, and applicable regulations on data protection and patient confidentiality. Data collection and reporting complied with the STROBE guidelines for observational studies and the EQUATOR Network recommendations.

## 3 Results

Eighteen patients with PG treated with selective IL‐23 inhibitors were included in the study (Table 1). The cohort comprised 11 women (61.1%) and 7 men (38.9%), all of Caucasian ethnicity. The mean age at disease onset was 46.2 ± 16.0 years, while the mean age at diagnosis was 50.3 ± 16.4 years, with a median diagnostic delay of 0.8 years (range 0.2–22.1).

**TABLE 1:** Baseline demographic and clinical characteristics of patients with pyoderma gangrenosum.

Variable	*n* = 18	Variable	*n* = 18
Sex, *n* (%)		*Comorbidities, n (%)*	
Female	11 (61.1)	Type II diabetes mellitus	5 (27.8)
Male	7 (38.9)	Arterial hypertension	8 (44.4)
Ethnicity, *n* (%)		Hypercholesterolemia	3 (16.7)
Caucasian	18 (100)	Peripheral arterial disease	3 (16.7)
Age at disease onset (years), mean (SD)	46.2 (16.0)	Chronic venous insufficiency	3 (16.7)
Age at diagnosis (years), mean (SD)	50.3 (16.4)	Deep vein thrombosis	4 (22.2)
Diagnostic delay (years), median (range)	0.8 (0.2–22.1)	Ischemic heart disease	2 (11.1)
PARACELSUS score, median (range)	15.5 (10–18)	Ulcerative colitis	3 (16.7)
Smoking status, *n* (%)		*Hematologic comorbidities*, *n (%)*	
Current smoker	2 (11.1)	Chronic lymphocytic leukemia	1 (5.6)
Former smoker	3 (16.7)	Hodgkin lymphoma	1 (5.6)
Never smoker	13 (72.2)	MGUS	1 (5.6)
Clinical variants of PG, *n* (%)		*Other comorbidities* ^a^	COPD, previous tuberculosis, pulmonary embolism, autoimmune thyroiditis (*n* = 2), polymyalgia rheumatica, hepatic steatosis (*n* = 2), metastatic breast cancer, previous breast cancer, neuroendocrine hepatic and pancreatic carcinoma, psoriasis, Dubowitz syndrome
Classical ulcerative	14 (77.8)		
Pustular/ulcerative	3 (16.7)		
Vegetative	1 (5.6)		

Abbreviations: COPD, chronic obstructive pulmonary disease; MGUS, monoclonal gammopathy of undetermined significance; SD, standard deviation.

^a^ Reported in single cases unless otherwise specified.

Metabolic and cardiovascular comorbidities were common, particularly arterial hypertension (44.4%) and type II diabetes mellitus (27.8%), along with dyslipidemia, peripheral arterial disease, chronic venous insufficiency, and thromboembolic or ischemic cardiovascular conditions. Inflammatory bowel disease (16.7%) and hematologic disorders (16.7%) were also frequently observed.

From a clinical standpoint, classic ulcerative PG represented the most common presentation, observed in 14 patients (77.8%), followed by pustular/ulcerative and vegetative variants in three (16.7%) and one (5.6%) cases, respectively.

All patients had previously received systemic corticosteroids (Table S2). Prior exposure to conventional immunosuppressants and biologic therapies was common, most frequently cyclosporine and anti–TNF‐α agents. All patients had highly refractory disease with prior exposure to multiple treatment lines (median 4, range 2–7).

Eleven patients (61.1%) were treated with guselkumab, six (33.3%) with risankizumab, and one (5.6%) with tildrakizumab (Table 2).

**TABLE 2:** Anti–IL‐23 treatment characteristics and baseline disease features.

Variable	*n* = 18
Anti–IL‐23 therapy, *n* (%)	
Guselkumab	11 (61.1)
100 mg at Weeks 0 and 4, then 100 mg every 8 weeks	5
100 mg at Weeks 0 and 4, then 100 mg every 4 weeks^a^	5
100 mg at Weeks 0 and 4, then 100 mg every 6 weeks	1
Risankizumab	6 (33.3)
150 mg at Weeks 0 and 4, then 150 mg every 12 weeks	1
150 mg at Weeks 0 and 4, then 150 mg every 8 weeks	2
150 mg at Weeks 0 and 4, then 150 mg every 4 weeks	1
300 mg at Weeks 0 and 4, then 300 mg every 12 weeks	2
Tildrakizumab	1 (5.6)
200 mg at Weeks 0 and 4, then 200 mg every 12 weeks	1 (5.6)
Status at follow‐up, *n* (%)	
Ongoing treatment	12 (66.7)
Lost to follow‐up	2 (11.1)
Death	1 (5.6)
Primary inefficacy	1 (5.6)
Secondary inefficacy	1 (5.6)
Adverse events	1 (5.6)
Ulcer characteristics	
Total number of ulcers, *n*	52
Ulcers per patient, median (range)	2 (1–12)
Ulcer location, *n* (%)	
Lower limbs	14 (77.8)
Back	3 (16.7)
Upper limbs	1 (5.6)
Breast	1 (5.6)
Lesion duration (years), median (range)	2.1 (0.5–23.1)
Total ulcer area (cm^2^), median (range)	95 (22–700)
Follow‐up duration, years, median (range)	0.97 (0.38–3.55)
Baseline characteristics	
Ulcer depth score, median (range)	3 (2–4)
Border/perilesional skin score, median (range)	3 (3–4)
Wound bed score, median (range)	3 (2–4)
IGAPg score, median (range)	3.5 (3–4)
Concomitant therapies, *n* (%)	
Systemic corticosteroids	16 (88.9)
Prednisone‐equivalent dose (mg/day), median (range)	32.5 (0–100)
Dapsone	1 (5.6)

^a^ One patient required dose escalation to guselkumab 200 mg every 4 weeks starting from Week 52.

Overall, 52 ulcerative lesions were evaluated, with a median of 2 lesions per patient (range 1–12) (Table 2). The most frequently involved anatomical sites were the lower limbs, affected in 14 patients (77.8%), followed by the trunk in 3 (16.7%), the breast in 1 (5.6%), and the upper limbs in 1 (5.6%). The median duration of ulcers at treatment initiation was 2.1 years (range 0.5–23.1 years). At baseline, the median total ulcer area was 95 cm^2^ (range 22–700). Median ulcer depth score was 3 (range 2–4), corresponding to lesions extending to the subcutaneous tissue or deeper. Median border/perilesional skin score was 3 (range 3–4), reflecting intense erythema with undermined or violaceous borders, while the median wound bed score was 3 (range 2–4), indicating diffuse to abundant exudate. Sixteen patients (88.9%) were receiving systemic oral corticosteroids at baseline, with a median daily prednisone‐equivalent dose of 32.5 mg (range 0–100). One patient (5.6%) was concomitantly treated with dapsone, which was discontinued 6 weeks later due to asthenia; no other concomitant immunosuppressive or immunomodulatory therapies specifically targeting PG were administered during IL‐23 inhibitor treatment.

At the time of analysis, 12 patients (66.7%) were continuing treatment with selective IL‐23 inhibitors and were under active clinical follow‐up (Table 2). Two patients (11.1%) were lost to follow‐up. One patient (5.6%) died during follow‐up due to hemorrhagic stroke, deemed unrelated to treatment. Treatment was discontinued in one patient (5.6%) because of primary inefficacy, in one (5.6%) because of secondary inefficacy, and in one (5.6%) due to myocardial ischemia, not considered drug‐related.

### 3.1 Total Ulcer Area

Total ulcer area progressively and significantly decreased during treatment with selective IL‐23 inhibitors (Table 3). Median ulcer area was 95 cm^2^ (range 22–700) at baseline and decreased to 70 cm^2^ (range 15–400) at 1 month, 40 cm^2^ (range 10–230) at 3 months, 25 cm^2^ (range 5–160) at 6 months, 12 cm^2^ (range 0–95) at 12 months, and 9 cm^2^ (range 0–88) at the last available observation.

**TABLE 3:** Longitudinal changes in clinical outcomes and corticosteroid dose during selective IL‐23 inhibition.

Total ulcer area (cm^2^)					
	*n*	Median	Range	Wilcoxon *W*	*p*
*Time point*					
Baseline	18	95	22–700	—	—
1 month	18	70	15–400	12.0	0.0069*
3 months	18	40	10–230	0.0	0.0003*
6 months	15	25	5–160	2.0	0.0002*
12 months	11	12	0–95	0.0	< 0.0001*
Last follow‐up	7	9	0–88	1.0	< 0.0001*
*Number of ulcers per patient*					
Baseline	18	2	1–12	—	—
1 month	18	2	1–13	34.0	0.41
3 months	18	1	0–11	8.0	0.004*
6 months	15	1	0–8	4.0	0.009*
12 months	11	0	0–4	1.0	0.005*
Last follow‐up	7	0	0–3	0.0	< 0.001*
*Ulcer depth score*					
Baseline	18	3	2–4	—	—
1 month	18	3	1–4	1.5	0.41
3 months	18	2.5	0–4	4.5	0.01*
6 months	15	2	0–3	0.0	0.003*
12 months	11	2	0–2	0.0	0.023*
Last follow‐up	7	1	0–2	0.0	0.046*
*Border/perilesional skin score*					
Baseline	18	3	2–4	—	—
1 month	18	3	1–4	1.5	0.41
3 months	18	2.5	0–4	4.5	0.01*
6 months	15	2	0–3	0.0	0.003*
12 months	11	2	0–2	0.0	0.023*
Last follow‐up	7	1	0–2	0.0	0.046*
*Wound bed score*					
Baseline	18	3	1–4	—	—
1 month	18	3	1–4	5.0	0.013*
3 months	18	2	0–3	0.0	0.007*
6 months	15	2	0–3	0.0	0.025*
12 months	11	1	0–2	0.0	0.004*
Last follow‐up	7	1	0–1	0.0	0.046*
*IGAPg score*					
Baseline	18	3.5	3–4	—	—
1 month	18	3	1–4	5.0	0.008*
3 months	18	2	0–3	0.0	0.004*
6 months	15	2	0–3	0.0	0.002*
12 months	11	1	0–2	0.0	0.012*
Last follow‐up	7	1	0–2	0.0	0.027*
*NRS pain*					
Baseline	18	8	3–10	—	—
1 month	18	5	1–9	10.0	0.004*
3 months	18	3	0–8	3.0	0.003*
6 months	15	2	0–6	0.0	< 0.001*
12 months	11	1	0–4	1.0	< 0.001*
Last follow‐up	7	0	0–4	0.0	< 0.001*
*Prednisone‐equivalent dose (mg/day)*					
1 month	18	32.34	0.00–88.39	—	—
3 months	18	20.83	0.00–71.43	3.0	0.002*
6 months	15	14.24	0.00–166.67	5.0	0.004*
12 months	11	0.00	0.00–25.09	8.0	0.009*
Last follow‐up	7	0.00	0.00–6.00	11.0	0.014*

Note:*p‐value statistically significant.

**FIGURE 1:**
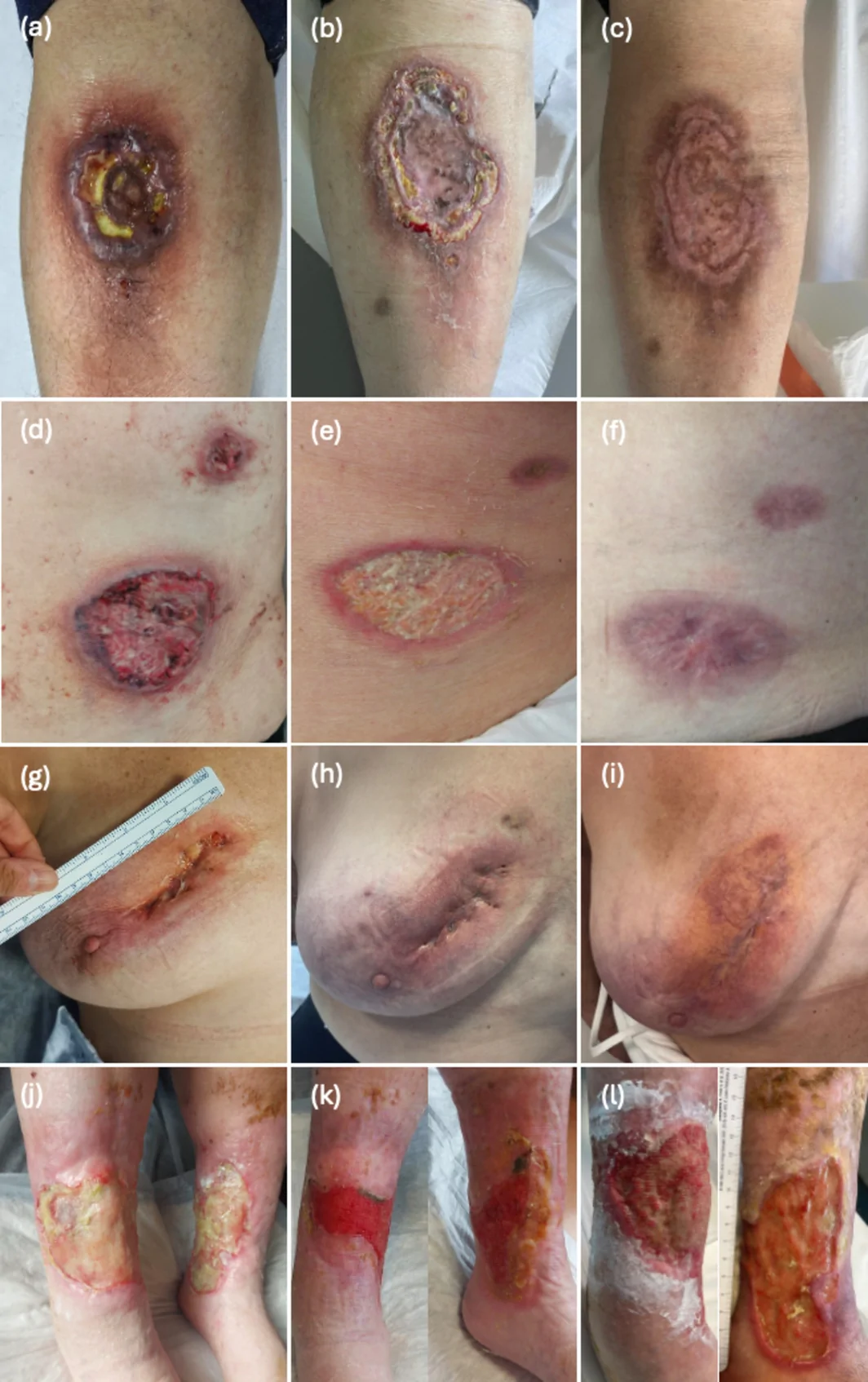
Clinical photographs at baseline (a, d, g, j), 12 (b, e, h, k), and 52 (c, f, i, l) weeks documenting progressive improvement in three patients (a–i) and secondary ineffectiveness in one case (j–l).

Global longitudinal analysis confirmed a statistically significant reduction in ulcer area over time (*p* < 0.001) (Figure 1). Pairwise comparisons showed a significant reduction already at 1 month compared with baseline (*p* = 0.0069), with further significant improvement at 3 months (*p* = 0.0003), 6 months (*p* = 0.0002), 12 months (*p* < 0.0001), and at the last available follow‐up (*p* < 0.0001).

### 3.2 Number of Active Ulcers

The median number of active ulcers per patient was 2 (range 1–12) at baseline and remained unchanged after 1 month of treatment (median 2; range 1–13; *p* = 0.41). From Month 3 onward, a significant reduction was observed, with a median of 1 active ulcer at 3 months (range 0–6; *p* = 0.004), 1 at 6 months (range 0–5; *p* = 0.009), and 0 at 12 months (range 0–3; *p* = 0.005), remaining stable at the last available observation (*p* < 0.001) (Table 3).

### 3.3 Ulcer Depth Score

Ulcer depth score showed a significant and progressive improvement over time (*p* < 0.001) (Table 3). The median ulcer depth score was 3 (range 2–4) at baseline and remained unchanged at 1 month (3 [range 1–4]; *p* = 0.41). A significant reduction was observed from 3 months onward, with median scores of 2.5 (range 0–4) at 3 months (*p* = 0.01), 2 (range 0–3) at 6 months (*p* = 0.003), and 2 (range 0–2) at 12 months (*p* = 0.023), reaching 1 (range 0–2) at the last available follow‐up (*p* = 0.046).

### 3.4 Border/Perilesional Skin Score

The median border score decreased from 3 (range 3–4) at baseline to 3 (1–3) at 1 month, 2 (0–3) at 3 months, 1 (0–3) at 6 months, 1 (0–2) at 12 months, and 0 (0–1) at the last available observation (Table 3).

The improvement was statistically significant over time (*p* < 0.001), with significant reductions versus baseline at all follow‐up visits (1 month *p* = 0.0008, 3 months *p* = 0.021, 6 months *p* = 0.005, 12 months *p* = 0.005, last follow‐up *p* = 0.034).

### 3.5 Wound Bed Score

Wound bed characteristics also improved significantly over time (*p* < 0.0001) (Table 3). Median wound bed score decreased from 3 (range 1–4) at baseline to 3 (range 1–4) at 1 month (*p* = 0.013), 2 (range 0–3) at 3 months (*p* = 0.007), 2 (range 0–3) at 6 months (*p* = 0.025), and 1 (range 0–2) at 12 months (*p* = 0.004), remaining stable at the last observation (median 1; range 0–1; *p* = 0.046), consistent with progressive re‐epithelialization.

### 3.6 IGAPg Score

IGAPg score showed significant and progressive improvement over time (*p* < 0.001) (Table 3). The median IGAPg score decreased from 3.5 (range 3–4) at baseline to 3 (range 1–4) at 1 month (*p* = 0.008). A significant improvement was observed from 3 months onward, with a median score of 2 (range 0–3) at 3 months (*p* = 0.004), 2 (range 0–3) at 6 months (*p* = 0.002), and 1 (range 0–2) at 12 months (*p* = 0.012), remaining stable at the last available observation (median 1; range 0–2; *p* = 0.027).

### 3.7 Pain Severity

Pain intensity significantly decreased during treatment (*p* < 0.001) (Table 3). Median NRS pain score decreased from 8 (range 3–10) at baseline to 5 at 1 month (*p* = 0.004), 3 at 3 months (*p* = 0.003), 2 at 6 months (*p* < 0.001), 1 at 12 months (*p* < 0.001), and 0 at the last available follow‐up (*p* < 0.001).

### 3.8 Prednisone‐Equivalent Dose

Systemic corticosteroid exposure showed a significant and progressive reduction over time (Table 3). The median prednisone‐equivalent dose was 32.34 mg/day (range 0.00–88.39) at 1 month. A statistically significant dose reduction was observed from 3 months onward, with a median dose of 20.83 mg/day (range 0.00–71.43) at 3 months (*p* = 0.002), 14.24 mg/day (range 0.00–166.67) at 6 months (*p* = 0.004), and complete discontinuation in most patients at 12 months (median 0.00 mg/day; range 0.00–25.09; *p* = 0.009), which was maintained at the last available follow‐up (median 0.00 mg/day; range 0.00–6.00; *p* = 0.014).

### 3.9 Comparative Analysis Between Guselkumab and Risankizumab

A comparative analysis was performed to explore potential differences in effectiveness between the two main selective IL‐23 inhibitors used in the cohort, guselkumab and risankizumab. As only one patient received tildrakizumab, the latter was excluded from the comparative analysis.

To account for heterogeneity in follow‐up duration and baseline ulcer burden, the percentage change in total ulcer area was calculated between baseline and the last available visit for each patient using a last observation data only (LODO) approach.

Patients treated with guselkumab showed a median percentage reduction in ulcer area of −99.2% (range −100% to 0%), whereas those treated with risankizumab showed a median reduction of −79.2% (range −100% to +5%). Mann–Whitney *U* test did not reveal a statistically significant difference between the two treatment groups (*p* = 0.182) (Table S3).

### 3.10 Univariate Analysis of Predictors of Clinical Response

Univariate analysis was conducted to investigate potential associations between clinical–demographic variables and the percentage reduction in ulcer area from baseline to LODO (Table 4).

**TABLE 4:** Univariate analysis of predictors of percentage ulcer area reduction.

Variable	*β* coefficient	95% CI	*t* value	*p*
Age	−0.42	−1.9 to 1.0	−0.47	0.64
Sex	3.5	−20.2 to 27.1	0.30	0.77
Body mass index (BMI)	−0.68	−2.4 to 1.1	−0.74	0.47
Current/former smoker	−9.8	−37.5 to 17.8	−0.74	0.47
Disease duration	−2.3	−7.4 to 2.8	−0.88	0.39
Baseline ulcer depth	−35.7	−69.3 to −2.1	−2.27	0.039*
Daily steroid dose (prednisone‐equivalent, mg)	0.10	−0.15 to 0.34	0.80	0.43
Diabetes mellitus	−14.5	−52.2 to 23.1	−0.83	0.42
Arterial hypertension	−10.1	−39.7 to 19.4	−0.74	0.47
Peripheral arterial disease	−18.9	−61.4 to 23.6	−0.93	0.37
Chronic venous insufficiency	−11.3	−44.8 to 22.3	−0.72	0.48
Number of previous treatment lines	−6.7	−16.2 to 2.8	−1.46	0.17

Note:*p‐value statistically significant.

Variables related to patient characteristics, disease features, and treatment‐related parameters were included in the analysis.

Baseline ulcer depth was the only factor significantly associated with percentage ulcer area reduction (*p* = 0.039), indicating that greater ulcer depth at treatment initiation was associated with a lower degree of ulcer area reduction. No other variables showed statistically significant associations with treatment response.

## 4 Discussion

In this multicenter, retrospective study, selective IL‐23 inhibition was associated with sustained clinical improvement in patients with refractory PG, including reductions in ulcer area and number, progressive improvement in ulcer characteristics, pain alleviation, and a meaningful steroid‐sparing effect. Clinical improvement was observed across multiple objective parameters. While ulcer area decreased early, more complex features such as ulcer depth and wound bed improved progressively over time, suggesting that structural healing may require longer exposure to IL‐23 inhibition. Overall disease severity, assessed by the IGAPg score, also improved significantly. Pain reduction further supports the impact of IL‐23 inhibition on both objective disease activity and patient‐reported outcomes. In exploratory analysis, guselkumab and risankizumab showed comparable efficacy, supporting a potential class effect of selective IL‐23 inhibition. Baseline ulcer depth emerged as the only predictor of response, consistent with the concept that advanced tissue damage may limit reversibility despite adequate inflammatory control.

Our findings are consistent with the emerging evidence on the off‐label use of selective IL‐23 inhibitors in PG (Table S4). Guselkumab, risankizumab, and tildrakizumab have been successfully employed in refractory PG, mainly in patients with long‐standing disease and multiple prior treatment failures. Early reports with guselkumab described rapid reductions in ulcer depth and exudation followed by complete re‐epithelialization within a few months, with sustained disease control and good tolerability [12, 13, 18]. Comparable clinical responses have been reported with risankizumab, including cases of recurrent and peristomal PG refractory to corticosteroids, immunosuppressants, and anti‐TNF‐α agents, achieving complete healing within 2–7 months [13, 15, 19, 20]. Similarly, tildrakizumab has been associated with near‐complete or complete ulcer resolution within 6–8 months in isolated cases of refractory PG [11, 21].

More recently, translational evidence has shown that IL‐23 inhibition is associated not only with clinical improvement but also with a reduction in dermal neutrophilic infiltrates and a downregulation of IL‐23p19 and IL‐17A expression in lesional skin, together with pain reduction and downregulation of inflammatory mediators at the systemic level [6]. The timing and durability of clinical responses reported in these studies closely mirror our observations, supporting the hypothesis that selective IL‐23 blockade may induce both early inflammatory control and a deeper immunomodulatory effect in refractory PG.

PG is driven by an autoinflammatory cytokine network in which the IL‐23/Th17/neutrophil axis plays a central role. IL‐23 overexpression at ulcer margins promotes Th17‐mediated production of IL‐17 and IL‐22, amplifying neutrophil recruitment, tissue damage, and impaired re‐epithelialization [6, 7]. Selective IL‐23p19 blockade inhibits this inflammatory cascade while preserving the IL‐12/IFN‐γ axis, resulting in reduced neutrophilic inflammation and ulcer healing [6, 14, 22]. The frequent association of PG with IL‐23–mediated systemic diseases further supports this targeted therapeutic approach [23]. The incomplete response may reflect the multifactorial pathogenesis of PG; therefore, combined targeted approaches acting on different inflammatory pathways could be considered in refractory cases [24].

### 4.1 Study Limitations

This study has several limitations. The small sample size reflects both the rarity of PG and the highly selected population, consisting of patients with severe, refractory disease and failure or contraindications to multiple prior systemic therapies. Although this represents a multicenter case series of PG treated with selective IL‐23 inhibitors, the absence of a control group limits definitive attribution of clinical responses to IL‐23 blockade.

It is important to acknowledge that the concomitant use of systemic corticosteroids during the early phases of treatment represents a potential confounding factor in the interpretation of the initial efficacy of IL‐23 inhibitors. In particular, the rapid anti‐inflammatory effects of corticosteroids may have partly contributed to the early reduction in ulcer activity observed during the first weeks of therapy—although it should be noted that all patients had prior failure to systemic corticosteroids alone. In contrast, the more sustained and stable clinical response documented over the mid‐ to long‐term follow‐up is likely attributable to the specific modulation of the IL‐23/Th17 axis. This temporal distinction between early anti‐inflammatory effects and long‐term disease control is crucial for interpreting the true therapeutic contribution of selective IL‐23 inhibition in refractory to conventional therapy PG.

Patient heterogeneity with respect to demographics, comorbidities, disease characteristics, prior treatments, and dosing regimens may have influenced response patterns but reflects real‐world clinical practice in PG. Despite standardized assessments performed according to Good Clinical Practice, some degree of interobserver variability cannot be excluded, and the lack of biological or histological endpoints limits mechanistic correlations.

## 5 Conclusions

This study adds real‐world data on the use of selective IL‐23 inhibitors in PG. Our findings support the potential role of the IL‐23/Th17 axis in PG pathogenesis and provide further clinical evidence for the tolerability of selective IL‐23 blockade, with encouraging signals of effectiveness in severe or relapsing disease.

Treatment resulted in sustained ulcer control, progressive re‐epithelialization, significant pain reduction, and a favorable safety profile with a relevant steroid‐sparing effect. These clinical outcomes are consistent with recent translational data, reinforcing the biological link between IL‐23 inhibition and resolution of chronic neutrophilic inflammation.

Despite the limitations inherent to the study design, the consistency of responses observed across centers and molecules strengthens the rationale for targeted use of IL‐23 inhibitors in patients with refractory PG or contraindications to traditional immunosuppressive therapies. Ongoing follow‐up and future prospective studies integrating clinical, histological, and molecular endpoints will be essential to better define long‐term outcomes, relapse risk, and predictors of response.

Overall, selective IL‐23 inhibition emerges as a rational and potentially disease‐modifying therapeutic strategy in PG, warranting further investigation in controlled prospective studies.

## Funding

This research was partially supported by the Italian Ministry of Health (Ricerca Corrente), Fondazione IRCCS Ca′ Granda Ospedale Maggiore Policlinico, Milan, Italy.

## Ethics Statement

This retrospective multicenter study was approved by the local Ethics Committee (approval number: 487_2020). The study was conducted in accordance with the principles of the 1964 Declaration of Helsinki and its later amendments, Good Clinical Practice guidelines, and applicable regulations on data protection and patient confidentiality.

## Consent

All patients had previously provided written informed consent for off‐label treatment and for the use of their anonymized clinical data during routine clinical practice. All patients provided written informed consent for publication of their anonymized clinical data and clinical photographs where applicable.

## Conflicts of Interest

The authors declare no conflicts of interest.

## Supporting information


**Table S1:** Clinical characteristics of pyoderma gangrenosum ulcers and scoring system.
**Table S2:** Previous systemic therapies.
**Table S3:** Comparison between guselkumab and risankizumab in percentage ulcer area reduction.
**Table S4:** Literature review of pyoderma gangrenosum treated with selective IL‐23 inhibitors.

## Data Availability

The data that support the findings of this study are available from the corresponding author upon reasonable request.
