# Supplementary material for: Selective IL‐23 Inhibition in Conventional Treatment‐Refractory Pyoderma Gangrenosum: A Multicenter, Retrospective Study
Source: Int J Dermatol. 2026 Apr 18;65(10):2055–65. doi: 10.1111/ijd.70430 (PMC13569361; doi:10.1111/ijd.70430)
Supplement: Supplementary file 1 [file IJD-65-2055-s001.docx]

**Table S1**. Clinical characteristics of pyoderma gangrenosum ulcers and scoring system.

| **Score** | **Ulcer depth** | **Ulcer border / perilesional skin** | **Wound bed** |
| --- | --- | --- | --- |
| 0 | Complete re-epithelialization / closed ulcer | No erythema; normal surrounding skin | Complete re-epithelialization / closed ulcer |
| 1 | Superficial erosion | Mild pink erythematous halo | Moist surface and/or crusts, no exudate |
| 2 | Dermal ulceration | Dull red erythema | Focal exudate |
| 3 | Deep ulcer extending to subcutaneous tissue | Intense bright red erythema and/or undermined border <50% | Diffuse exudate |
| 4 | Exposure of muscle, tendon, or bone | Violaceous or grayish erythema and/or undermined border 50–100% and/or bullous detachment | Abundant, diffuse exudate, possibly extending beyond ulcer margins |

**Table S2**. Previous systemic therapies.

| **Variable** | **n=18** |
| --- | --- |
| **Previous therapies, n (%)** |  |
| Systemic corticosteroids | 18 (100) |
| Cyclosporine | 7 (38.9) |
| Azathioprine | 1 (5.6) |
| Methotrexate | 1 (5.6) |
| Adalimumab | 10 (55.6) |
| Infliximab | 6 (33.3) |
| Dapsone | 4 (22.2) |
| Certolizumab pegol | 1 (5.6) |
| Ixekizumab | 1 (5.6) |
| Secukinumab | 1 (5.6) |
| Bimekizumab | 2 (11.1) |
| Anakinra | 1 (5.6) |
| Baricitinib | 1 (5.6) |

**Table S3**. Comparison between guselkumab and risankizumab in percentage ulcer area reduction.

| **Treatment** | **n** | **Median ulcer area reduction (%)** | **Range (%)** | **Mann–Whitney U** | **p value** |
| --- | --- | --- | --- | --- | --- |
| Guselkumab | 11 | −99.2 | −100 to 0 | 19.5 | 0.182 |
| Risankizumab | 6 | −79.2 | −100 to +5 |  |  |

**Table S4**. Literature review of pyoderma gangrenosum treated with selective IL-23 inhibitors.

| **Author (Year)** | **Drug** | **Age / Sex** | **Lesion location** | **Treatment regimen** | **Clinical outcome** | **Time to response** |
| --- | --- | --- | --- | --- | --- | --- |
| Burgdorf et al. (2020) [15] | Risankizumab | 59 / F | Lower limb | 150 mg every 12 weeks | Complete healing | ~7 months |
| Weigelt & Kirsner (2021) [19] | Risankizumab | 43 / F | Peristomal | 150 mg every 12 weeks | Complete healing | 2 months |
| Orita et al. (2022) [18] | Risankizumab | 68 / M | Shoulder | 150 mg every 12 weeks | Complete healing | 3 months |
| Michelucci et al. (2024) [14] | Risankizumab | 40, 50 / F, F | Lower limbs | 150 mg at weeks 0 and 4, then every 10 weeks | Complete healing | 4–6 months |
| Baier & Barak (2021) [12] | Guselkumab | 60 / F | Left leg | 100 mg every 8 weeks | Complete healing | 6 months |
| Reese et al. (2022) [13] | Guselkumab | 49 / F | Lower limb | 200 mg every 8 weeks | Complete healing | 4 months |
| Botvid et al. (2024) [17] | Guselkumab | 35, 39 / F, F | Peristomal, lower limbs | 400 mg every 6–7 weeks | Complete healing | 6–12 months |
| John & Sinclair (2019) [20] | Tildrakizumab | 69 / M | Penis | 100 mg at weeks 0 and 4, then every 12 weeks | Complete healing | 8 months |
| Leow & Zubrzycki (2022) [11] | Tildrakizumab | 85 / F | Left leg | 100 mg at weeks 0 and 4, then every 8–6 weeks | Complete healing | 6 months |
| Flora et al. (2025) [6] | Tildrakizumab | 8 patients (45–74, M/F) | Lower limbs and mixed sites | 100 mg at weeks 0 and 4, then every 12 weeks | Clinical improvement | ~3 months |
